# Real-time mechanism-based interventions for daily alcohol challenges: Protocol for ecological momentary assessment and intervention

**DOI:** 10.1177/20552076241311731

**Published:** 2025-01-22

**Authors:** Shuyan Liu, Matthias Haucke, Rika Groß, Kay Schneider, Jaekyung Shin, Fabian Arntz, Patrick Bach, Tobias Banaschewski, Christian Beste, Lorenz Deserno, Ulrich Ebner-Priemer, Tanja Endrass, Marvin Ganz, Ali Ghadami, Marco Giurgiu, Andreas Heinz, Falk Kiefer, Reinhold Kliegl, Bernd Lenz, Marta Anna Marciniak, Andreas Meyer-Lindenberg, Andreas B. Neubauer, Michael Rapp, Michael N. Smolka, Jens Strehle, Rainer Spanagel, Gianna Spitta, Heike Tost, Henrik Walter, Hilmar Zech, Dominic Reichert, Markus Reichert

**Affiliations:** 1 Department of Psychiatry and Psychotherapy (Campus Charité Mitte), 14903 Charité—Universitätsmedizin Berlin, Berlin, Germany; 2 German Center for Mental Health (DZPG), Partner Sites Berlin/Potsdam and Heidelberg/Mannheim/Ulm, Germany; 3 Department of Psychiatry and Psychotherapy, Central Institute of Mental Health, Medical Faculty Mannheim/Heidelberg University, Mannheim, Germany; 4 Department of Sports and Health Sciences, 26583 University of Potsdam, Potsdam, Germany; 5 Department of Addictive Behavior and Addiction Medicine, 27188 Central Institute of Mental Health (CIMH), Medical Faculty Mannheim, Heidelberg University, Mannheim, Germany; 6 Department of Child and Adolescent Psychiatry and Psychotherapy, 27188 Central Institute of Mental Health, Medical Faculty Mannheim, 9144 Heidelberg University, Mannheim, Germany; 7 University Neuropsychology Center (UNC), 9169 TU Dresden, Dresden, Germany; 8 Department of Child and Adolescent Psychiatry, Psychotherapy and Psychosomatics, University Hospital and 9190 University Würzburg, Wurzburg, Germany; 9 Addiction Research, Institute for Clinical Psychology and Psychotherapy, Faculty of Psychology, Technische Universität Dresden, Dresden, Germany; 10 Institute of Sports and Sports Science, 150232 Karlsruhe Institute of Technology, Karlsruhe, Germany; 11 Healthy Longevity Center, University of Zurich, Zurich, Switzerland; 12 Department of Psychology, Education and Child Studies, 6984 Erasmus University Rotterdam, Rotterdam, The Netherlands; 13 9165 RWTH Aachen University, Aachen, Germany; 14 Center for Information Services and High Performance Computing (ZIH), 9169 Technische Universität Dresden, Dresden, Germany; 15 Institute of Psychopharmacology, 27188 Central Institute of Mental Health, Medical Faculty Mannheim, 9144 Heidelberg University, Mannheim, Germany; 16 Department of eHealth and Sports Analytics, Faculty of Sport Science, 9142 Ruhr-University Bochum, Bochum, Germany; 17 Department for Sport and Exercise Science, Paris Lodron University Salzburg, Salzburg, Austria

**Keywords:** Alcohol consumption, loneliness, craving, real-life assessment and intervention, cognitive reappraisal, physical activity

## Abstract

**Background:**

Advancing evidence-based, tailored interventions for substance use disorders (SUDs) requires understanding temporal directionality while upholding ecological validity. Previous studies identified loneliness and craving as pivotal factors associated with alcohol consumption, yet the precise directionality of these relationships remains ambiguous.

**Objective:**

This study aims to establish a smartphone-based real-life intervention platform that integrates momentary assessment and intervention into everyday life. The platform will explore the temporal directionality of contextual influences on alcohol use among individuals experiencing loneliness and craving.

**Methods:**

We will target 180 individuals aged 18 to 70 in Germany who report loneliness, alcohol cravings, and meet risk or binge drinking criteria (over 14 standard drinks per week or five drinks in a single day for males, and over seven drinks per week or four drinks in a single day for females). Using a Within-Person-Encouragement-Design and Just-In-Time-Adaptive-Interventions, we will manipulate the contexts of loneliness and alcohol craving with cognitive reappraisal and physical activity interventions against a control condition (working memory task).

**Results:**

Recruitment started in June 2024, with data collection and processing expected by June 2027.

**Conclusion:**

Our real-life intervention platform endeavors to serve as a robust tool for discerning the directionality of the effects from time series data in everyday life influences on alcohol use for the future study. Ultimately, it will pave the way for low-threshold prevention, clinical treatment, and therapy to target diverse contexts of everyday life in SUD.

**Trial registration:**

German Clinical Trials Register DRKS00033133.

## Introduction

The desire for social connection is one reason people consume substances such as alcohol and cannabis.^1,2^ Increased substance use can heighten the risk for substance abuse and dependence. In Germany, up to 19% of the population engage in hazardous drinking.^3^ Previous studies have identified loneliness and craving as significant factors associated with alcohol consumption. However, the precise temporal direction of these relationships—critical for establishing causality—remains unclear.^4,5^ To ascertain the temporal direction of these contextual correlates for real-life treatment targets, adopting an ecological approach to momentary intervention is required. Therefore, the objective of this study is to establish a real-life intervention platform aimed at testing temporal directionality through experimental manipulation in everyday life, particularly in the context of loneliness and alcohol craving.

Loneliness, as the distressing experience of a discrepancy between one's desired and actual social connection,^6^ correlates with adverse health behaviors such as increased alcohol use and physical inactivity.^2^ A Eurofound survey conducted in 2016, prior to the pandemic, revealed that 12% of EU citizens experienced loneliness more than half of the time. This figure doubled to 25% during the first COVID-19 outbreak, with an additional 15.5% of individuals reporting feeling lonely more than half of the time.^7^ Loneliness had a strong effect on alcohol consumption among alcohol use disorder (AUD) participants across age groups.^4^ In particular, the sequential latent class regression models in our previous study showed that loneliness predicted change in alcohol consumption patterns over time in subsamples including adolescents and young adults, and middle-aged men.^8^

Craving, as “an intense desire or urge” for substances, is described as a consequence of the conflict between the need to consume substance and the desire to abstain; it is recognized as one cardinal symptom of AUD and serves as a major relapse predictor.^9^ Craving is not only an established marker for diagnoses on a cross-sectional and retrospective assessment level, but also evidenced to correlate with substance use on a within-subject and momentary level in everyday life: the more a person craves a substance at a given moment, the higher the consumption is, and vice versa.^1^ Recent studies argue for craving to be a promising causal marker with prognostic value and high potential as a treatment target for interventions triggered by ecological momentary assessment (EMA).^10^

The EMA methodology (e.g. smartphone-based electronic diaries and wearables) enables to investigate intra- and interindividual effects as well as reciprocal relationships.^11,12^ EMA involves repeatedly sampling of individuals’ behaviors and experiences in real-time within their natural environments. This method minimizes recall bias and enhances ecological validity, while approximating temporal dynamics (e.g. Granger causality).^13^ However, EMA can also impose a burden on participants, requiring them to respond to repeated questionnaires throughout the day, which may disrupt ongoing activities and cause distress.^14^ We can apply EMA to gather intensive longitudinal data (ILD) on determinants and consequences of drug intake.^15^ On the one hand, ILD is more ecologically valid, as it directly measures the subjects’ experience in their current environment; on the other hand, it allows to approximate temporal associations. However, causal inferences can only be established if: (1) variables of interest are correlated with each other, (2) a temporal order is given, (3) hidden third variables are controlled or explained; and (4) the direction of the effect is indispensable (e.g. heightened levels of craving usually correspond to increased alcohol consumption).^16^ Accordingly, experimental designs enable to test for causality through experimental manipulation under laboratory conditions and control of potential hidden influences.^17^

To approximate causality and test temporal directionality underlying drug intake in everyday life, we need to combine the advantages of laboratory and ambulatory approaches. This combination allows us to achieve both ecological validity and approximate causal temporal inferences (i.e. an ecological approach to intervention). EMA enables the collection of time series data, which can be used to approximate the temporal necessity of causality, known as Granger causality (i.e. where the cause precedes the effect).^18^

Just-In-Time-Adaptive-Interventions (JITAIs)^19^ are recognized as a first key step toward approximating causality within subjects in everyday life.^20^ In essence, JITAIs use real-time analyses to determine momentary states (e.g. EMA of loneliness and alcohol craving and device-based read-outs, such as acceleration signals for physical activity detection), and controlled or randomized intervention-assignments (e.g. an instruction to engage in physical activity after a couple of high-craving ratings). However, experimental manipulations may be infeasible in everyday life due to practical or ethical reasons (e.g. participants may be unable to interrupt their task to follow intervention-instructions under high work-related stress).

Here, the Within-Person-Encouragement-Design (WPED)^21^ offers a viable solution through three key elements: (1) smartphone-based, randomly assigned encouragement to engage in an intervention as an instrumental variable (Encouragement), (2) the intervention (i.e. cognitive reappraisal, physical activity) itself (Treatment), (3) target variables (i.e. loneliness and alcohol craving) that are to be causally influenced by the Treatment (Outcome). Based on an instrumental variable estimation approach, a causal treatment effect on the outcome can be analyzed if a correlation between the encouragement and the treatment is present and given the theoretical assumption that any encouragement effects on the outcome are fully mediated by the treatment. The WPED combines three major approaches: (1) multilevel analyses of within-subject mechanisms, (2) experimental within-subject manipulation of the “treatment” variable, and (3) randomized encouragement as an instrumental variable to induce exogenous experimental variation if consistent adherence to the “treatment” is not realistic.

Both cognitive reappraisal and physical activity have been associated with reduced loneliness and alcohol craving.^22–26^ At the behavioral and neurobiological levels, both can improve cognition functions.^27,28^ However, they are very different in nature: on the one hand, cognitive reappraisal is a collection of “structured, goal-directed, and collaborative intervention strategies that focus on the exploration, evaluation, and substitution of the maladaptive thoughts, appraisals, and beliefs that maintain psychological disturbance.”^29^ In the context of AUD, at low engagement in reappraisal, greater startle reactivity to uncertain threat was associated with greater problem alcohol use.^30^

On the other hand, physical activity describes a broad wealth of human movements ranging from distinct types of exercise (e.g. dancing, jogging as a structured physical activity characterized by high energy expenditure and prolonged duration for a fitness goal) to different nonexercise activities (NEAs; i.e. all other daily physical activities including walking and stair-climbing, or sedentary breaks such as short-term movement programs), with the latter NEA especially qualifying for JITAI served by smartphone applications.^31,32^ The type, duration, and intensity of physical activity can be tailored toward individual preferences and improvements of specific symptoms. Physical activity is a promising adjunctive treatment for substance use disorder (SUD)^33^ and benefits SUD patient care^34^; e.g. it has been associated with reductions in craving across different substances (e.g. tobacco, marijuana, alcohol), and especially in AUD, several studies indicate that physical activity can decrease craving for alcohol.^35^ In particular, the recent literature assumes that “craving could be specifically targeted by short and easily achievable physical exercise” and short bouts of moderate physical activity such as brisk walking are correlated with reduced craving.^36^

**Figure 1.**
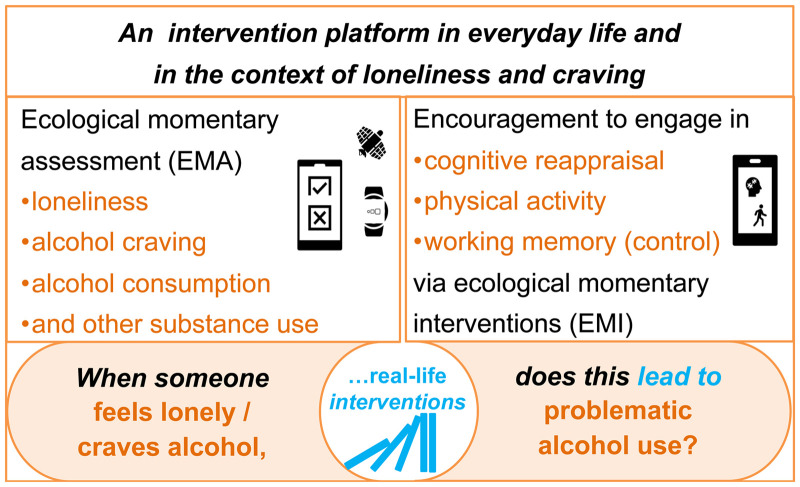
Schematic overview of daily life causality testing in our study. Systematic experimental manipulation in phases of momentary loneliness/alcohol craving levels through the incorporation of cognitive reappraisal (Intervention 1), physical activity (Intervention 2), and working memory (control condition) via interactive smartphone triggers will allow to test for the (non)causal nature of real-life correlates (e.g. loneliness and alcohol craving) of alcohol intake.

Therefore, given both these proven benefits of cognitive reappraisal and physical activity for SUD prevention and treatment as outlined above, shown in Figure 1, and their high suitability for momentary application in everyday life (e.g. short yet effective cognitive reappraisal and physical activity programs guided via smartphone application), they qualify as highly promising tools and interventions in intensive longitudinal research for experimental manipulation in everyday life to elucidate the real-life causality of change among individuals with SUDs.^37,38^ Depending on the momentary settings (e.g. in work or leisure time) and personal preferences as well as momentary cognitive capacities, either the cognitive reappraisal or the physical activity intervention may be better suited to, for example, reduce momentary loneliness and alcohol craving.

Therefore, the overarching aim of our study is to establish a real-life intervention platform that incorporates a JITAI^39^ into everyday life to test the temporal direction of factors that lead to substance use. Our approach positions us uniquely to: (1) understand the temporal directionality of mechanisms of substance use in everyday life, (2) set the evidence-basis for an increasing number of (digital) mental health interventions designed for substance use through separating disease causes from consequences, (3) guide JITAIs^19^ for the right type/amount/time of support by adapting to individuals’ changing internal and contextual states.

We hypothesize (H1) that cognitive reappraisal and physical activity interventions will reduce momentary loneliness (H1a) and/or alcohol craving (H1b). In addition, we assume (H2) that decreases in momentary loneliness and/or alcohol craving will correspond to a decrease in real-life alcohol consumption.

## Methods

### Participants and recruitment

The study will be conducted between June 2024 and June 2027 in Germany. It is a part of the transregio (TRR 265) project “Addiction research consortium: Losing and regaining control over drug intake” in Germany.^40^ The TRR265 cohort targets 900 people with mainly mild to moderate AUD and 150 age-matched controls. To test causality in real-life mechanisms of problematic alcohol use, we will target 180 risk and/or binge drinkers aged 18 to 70 who report feeling lonely and are craving alcohol. Exclusion criteria include chronic obstructive pulmonary disease, coronary heart disease, heart failure, a body mass index of ≥35 or ≤18, use of assistive devices like walkers, and pregnancy. The target sample will be recruited and screened both from TRR 265 cohort and the general population in Germany. With the assistance of the professional recruitment agencies, we will deploy advertisements across multiple media channels, including newspapers, social media platforms, notice boards, and within both inpatient and outpatient clinics. Participants will receive a monetary reward as compensation after completing the study. The amount of compensation is determined by a percentage of the completed study items.

Risky alcohol use and binge drinking are defined by the German Federal Centre for Health Education (Bundeszentrale für gesundheitliche Aufklärung, BZgA),^41^ and the German Ministry of Health.^42^ For men, binge drinking is defined as consuming five or more drinks on a single day, while risk drinking is exceeding 14 drinks per week over the past 30 days. For women, binge drinking is consuming four or more drinks on a single day, and risk drinking is exceeding seven drinks per week within the past 30 days. For calculating alcohol consumption, we will ask participants for their sex assigned at birth. Participants will be presented with a list of common alcoholic beverages,^43^ and will be asked to report the number of each type of beverage they consume on a weekly basis.^44^

For screening purposes, high trait loneliness will be assessed by using the short eight-item form UCLA Loneliness Scale (ULS-8; cutoff scores ≥ 16).^45^ High trait alcohol craving will be assessed by using the five-item Penn Alcohol Craving Scale (PACS-5; cutoff ≥ mean from a pilot sample of 50 participants).^46^ We will also assess their sociodemographic characteristics (i.e. age, gender, education level, and income), alcohol consumption, mental health status, and cognitive performance.

### Ethics and informed consent

The study will be conducted in accordance with the Helsinki Declaration of 1975 and the ethics committee's standards. The study was evaluated by the Ethics Committee at Charité—Universitätsmedizin Berlin (reference number: EA1/270/22) and at Heidelberg University (reference number: 2023-536). Written informed consent will be obtained from all participants before they start the study.

### Sample size and power considerations

Our power estimation is based on simulation studies for the WPED.^21^ We expect a compliance rate exceeding 70% with three (non)encouragements provided daily. Previous research indicates that average compliance rates in similar studies can reach up to 84%. However, many studies with lower compliance rates often remain unpublished.^47^ In addition, earlier studies report compliance rates ranging from 50% to 90%.^48^ Given this variability, we have adopted a balanced assumption of a 70% compliance rate. In addition, the dropout rate for technological-based psychological intervention varies widely, from 2% to 83%.^49^ We also take a balanced approach by assuming a 20% dropout rate.

The study will involve 21 intervention days, resulting in a maximum of 63 intervention data points per person. At a total recruitment of 180 participants and a resulting sample size of *n* = 144 participants subsequent to a 20% drop-out would result in a total of 6350 data points at a compliance of 70% (144 participants × 21 intervention days × 3 (non)encouragements × 0.7 compliance rate). According to Schmiedek and Neubauer's power simulation studies for WPEDs,^21^ our sample size will allow for sufficient power to detect a small- to medium-sized average treatment effect across a broad range of conditions.

### Design and procedure

To design the real-life intervention platform, we will combine JITAIs as smartphone applications (apps) with EMA (including physical activity tracking via accelerometers and electronic diaries). For this purpose, we will use the software *movisensXS* and *TherapyBuilder* (both *movisens* GmbH, Karlsruhe) to incorporate two JITAIs including cognitive reappraisal and physical activity, and an active control condition involving a working memory task.^50^ In particular, we will generate motivators to break up sedentary phases and to increase the number of steps taken.

**Figure 2.**
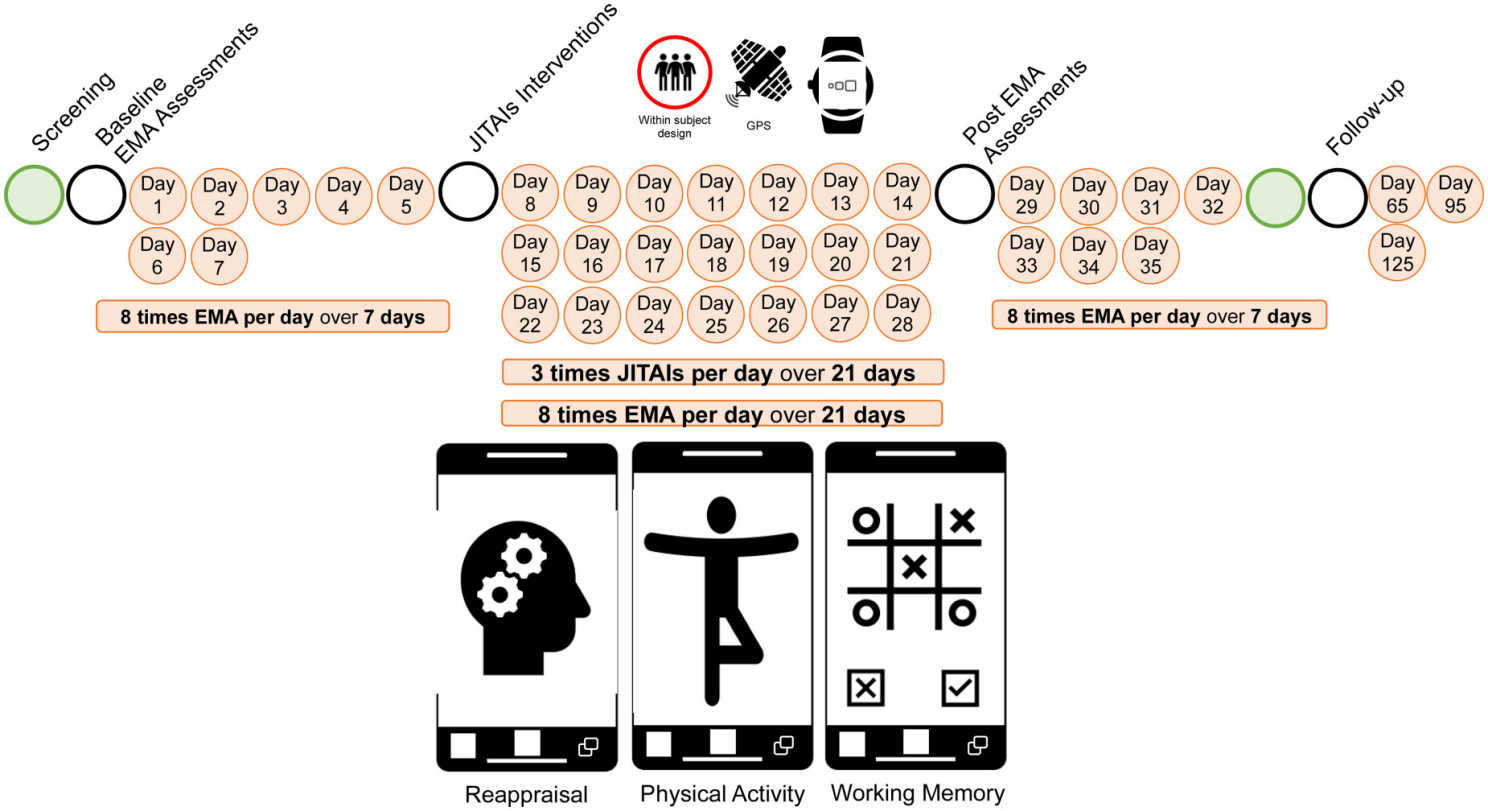
Experimental design. After initial screening and baseline measurements, a 1-week preintervention ambulatory assessment procedure will be followed by 3 weeks of WPED including JITAIs and subsequent 1-week postintervention EMA. Follow-up measurements will be conducted 12 weeks after the EMA procedure. WPED: within-person-encouragement-design; JITAI: Just-In-Time-Adaptive-Intervention; EMA: ecological momentary assessment.

We will employ a WPED with counterbalancing. All participants will take part in three conditions in a counterbalanced order, with each participant randomly assigned to receive one of the following: Intervention 1, involving cognitive reappraisal; Intervention 2, which includes physical activity; or an active control condition featuring a gamified working memory task. The duration of each condition will be approximately 6 min. All participants will start with a baseline EMA for seven consecutive days (eight times per day), followed by a 21-day JITAI (three times per day), while also receiving the usual EMA eight times per day. They will end up with a follow-up EMA for seven consecutive days (eight times per day; see Figure 2).

Two fixed-time EMAs will be administered daily: the first in the morning at 08:00 and the last in the evening at 22:00. The morning EMA will include questions about sleep quality.^51^ During the evening EMA, participants will complete a brief 3-min gamified response inhibition task,^50^ developed based on the widely used stop signal task. This task is designed to assess potential changes in cognitive functions over time and examine the mediation effects of JITAI on enhancing cognitive function in relation to problematic alcohol use. In addition to two fixed EMAs, eight randomly timed EMAs will be send each day between 08:00 and 21:00, with at least a 60-min interval between them. Participants can delay their responses to the EMAs by up to 60 min to ensure data collection, especially if they are in situations where using their phone is not feasible. If a participant does not engage with the intervention within the 60-min window, we will still send a questionnaire asking about their experienced loneliness and craving, and reasons for skipping the intervention.

**Figure 3.**
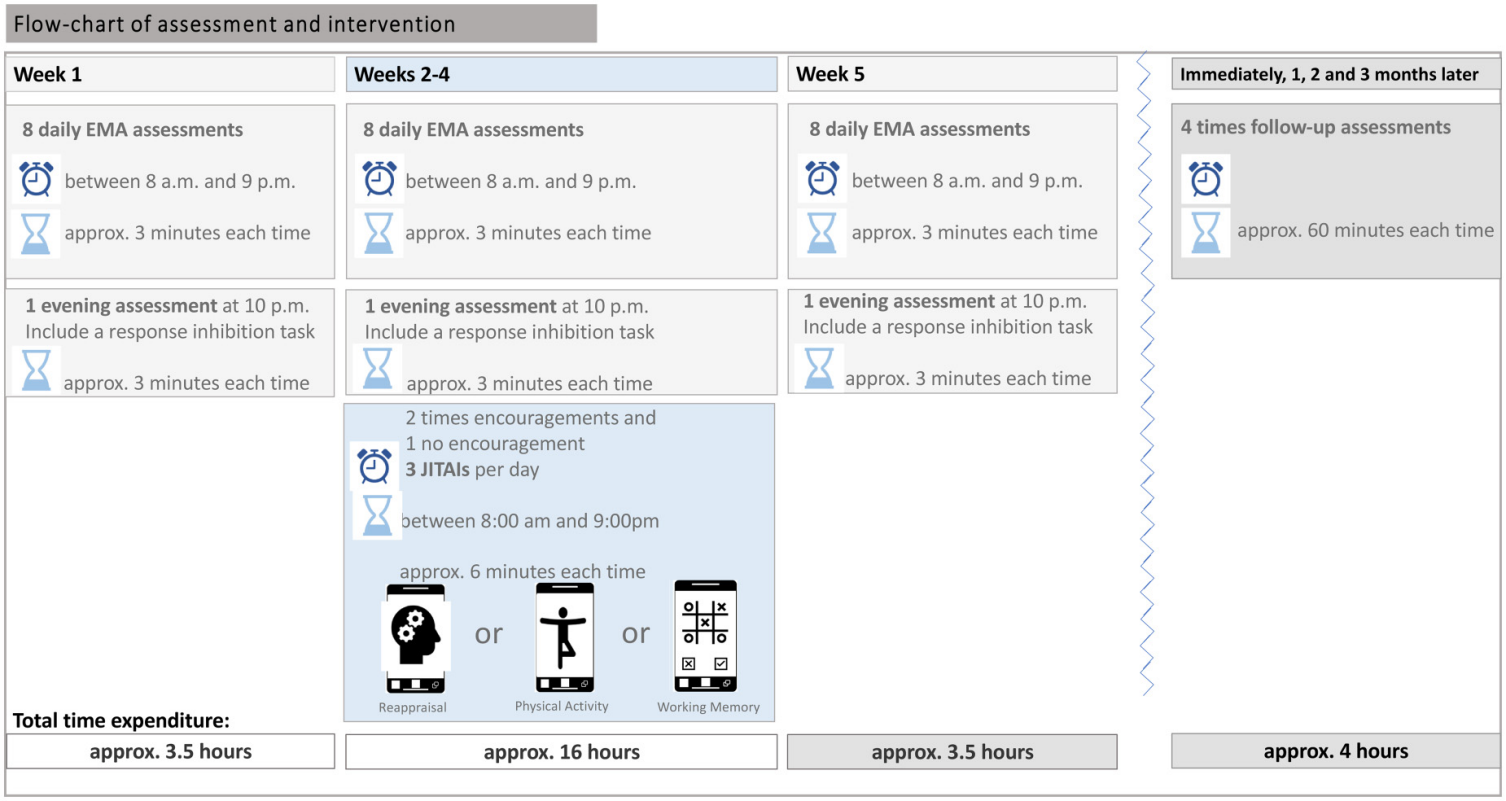
Flow-chart of assessment and intervention.

At the end of the 21-day JITAI study, we will evaluate participants’ perceptions of the interventions using the Acceptability E-scale^52^ the Digital Working Alliance Inventory,^53^ and a modified six-item Intervention-elicited Reactance Scale.^54^ Moreover, to evaluate the long-term effects, we will conduct follow-up assessments three times 1 month apart (i.e. 1 month, 2 months, and 3 months later). Figure 3 provides a graphical overview of the project.

**Figure 4.**
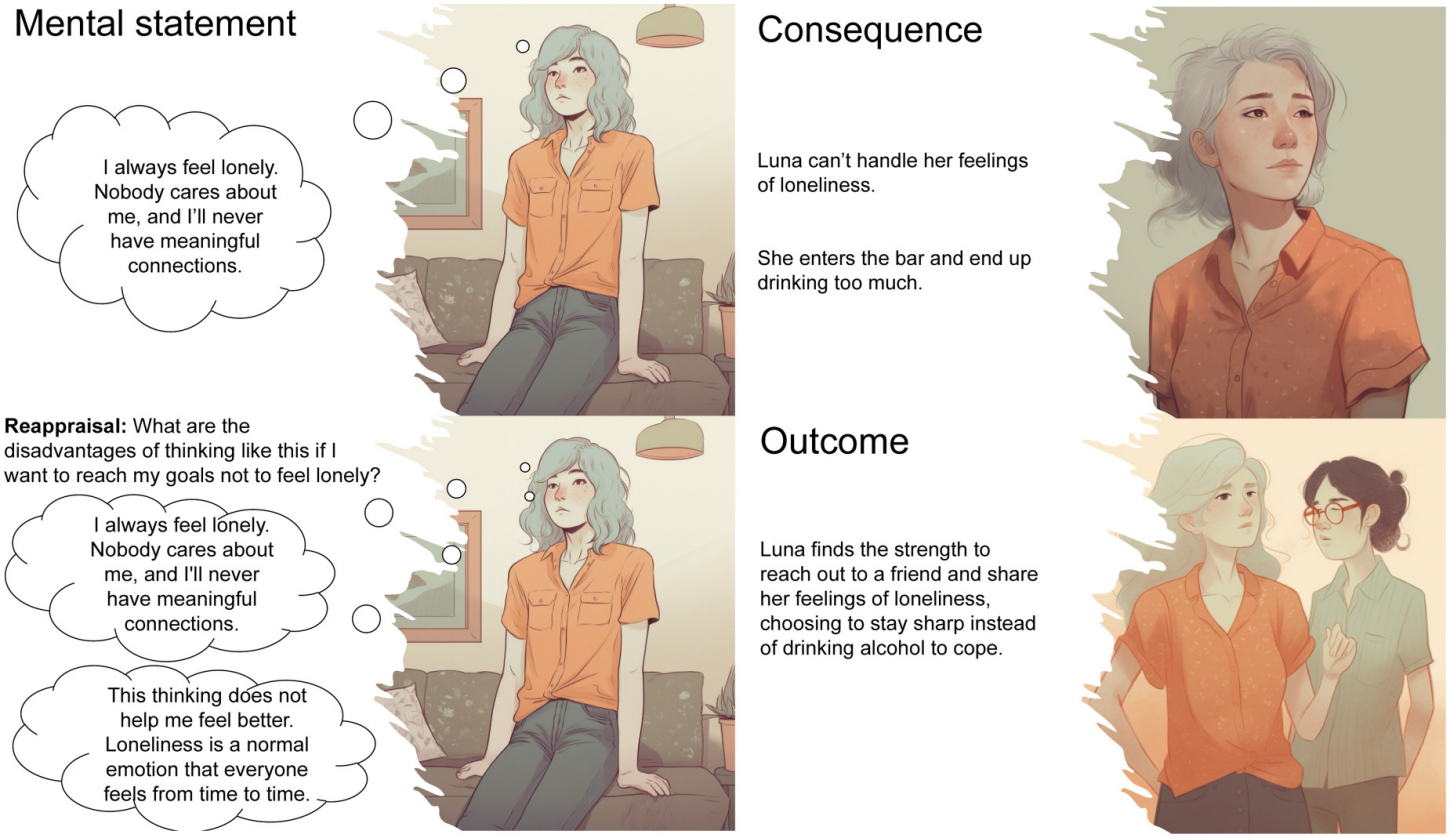
Illustrative screenshots from the comic elucidating cognitive reappraisal. This comic was created by us using the AI image generator Midjourney.

*Intervention 1 (cognitive reappraisal)*. Cognitive reappraisal describes the process of actively challenging (reappraising) maladaptive beliefs and thoughts associated with distressing mental states, such as loneliness and drug craving.^55–59^ In an initial laboratory session lasting approximately 20–30 min, participants will receive an explanation of cognitive reappraisal and be prompted to identify and challenge thoughts (reappraise) associated with cravings and loneliness. To illustrate the concept of cognitive reappraisal, we developed a comic featuring male and female characters grappling with loneliness and alcohol cravings. This comic was created using the AI image generator Midjourney (see Figure 4). We have a subscription to Midjourney and created Figure 4 on our own using the Midjourney. Participants can access a video illustrating the reappraisal techniques to refresh their understanding of how reappraisal functions (https://www.trr265.org/en/domäne-c05-video).

The JITAI will take approximately 6 min. It will begin by prompting participants to describe the situations in which they encountered feelings of loneliness or cravings. Subsequently, they will be asked to articulate the thought that most triggers their loneliness or craving. Finally, participants will be tasked with documenting how they challenge this thought, employing four reappraisal strategies.^60^ (1) Considering disadvantages: What are the disadvantages of thinking like this if I want to reach my goals to drink less or not to feel lonely. (2) Checking reality: Have I had any experiences to show that this mental statement is not always true? (3) Use other people as a reference point: What would someone who knows to handle this situation well say if they knew I was trying to reduce my drinking/not to feel lonely? (4) Imagine giving advice: What advice could you give to a close person in a similar situation? After each reappraisal, participants will be asked to rate their conviction regarding the thought related to loneliness or craving on a horizontal Visual Analog Scale (VAS)^61,62^ ranging from 0 (“not convinced at all”) to 10 (“strongly convinced”). In addition, they will be prompted to assess the difficulty of reappraising the specific situation using a horizontal VAS ranging from 0 (“strongly disagree”) to 10 (“strongly agree”).

*Intervention 2 (physical activity)*. The physical activity intervention will be designed to increase participants’ step count within a set timeframe. Previous studies have been found that moderate-intensity exercise sessions lasting 5 to 15 min can significantly reduce alcohol cravings.^36,63–65^ Accordingly, our physical activity intervention encourages participants to take a brisk 6-min walk (or engage in indoor walking). During this activity, the number of steps per minute is tracked using a *movisens* Move4 wrist-sensor, providing participants with real-time feedback on their walking pace. Control measures involve automatically assessing the number of steps taken during exercise using the *movisens* Move4 activity sensor.

*Active control (a gamified working memory task)*. The working memory is facilitated by the Great Brain Experiment app,^66^ which has been tested and validated in German.^50^ The task is gamified to enhance participant engagement and is designed based on a widely used delay-match-to-sample task.^67^ For more detailed description of the task, see Brown et al.^66^

**Figure 5.**
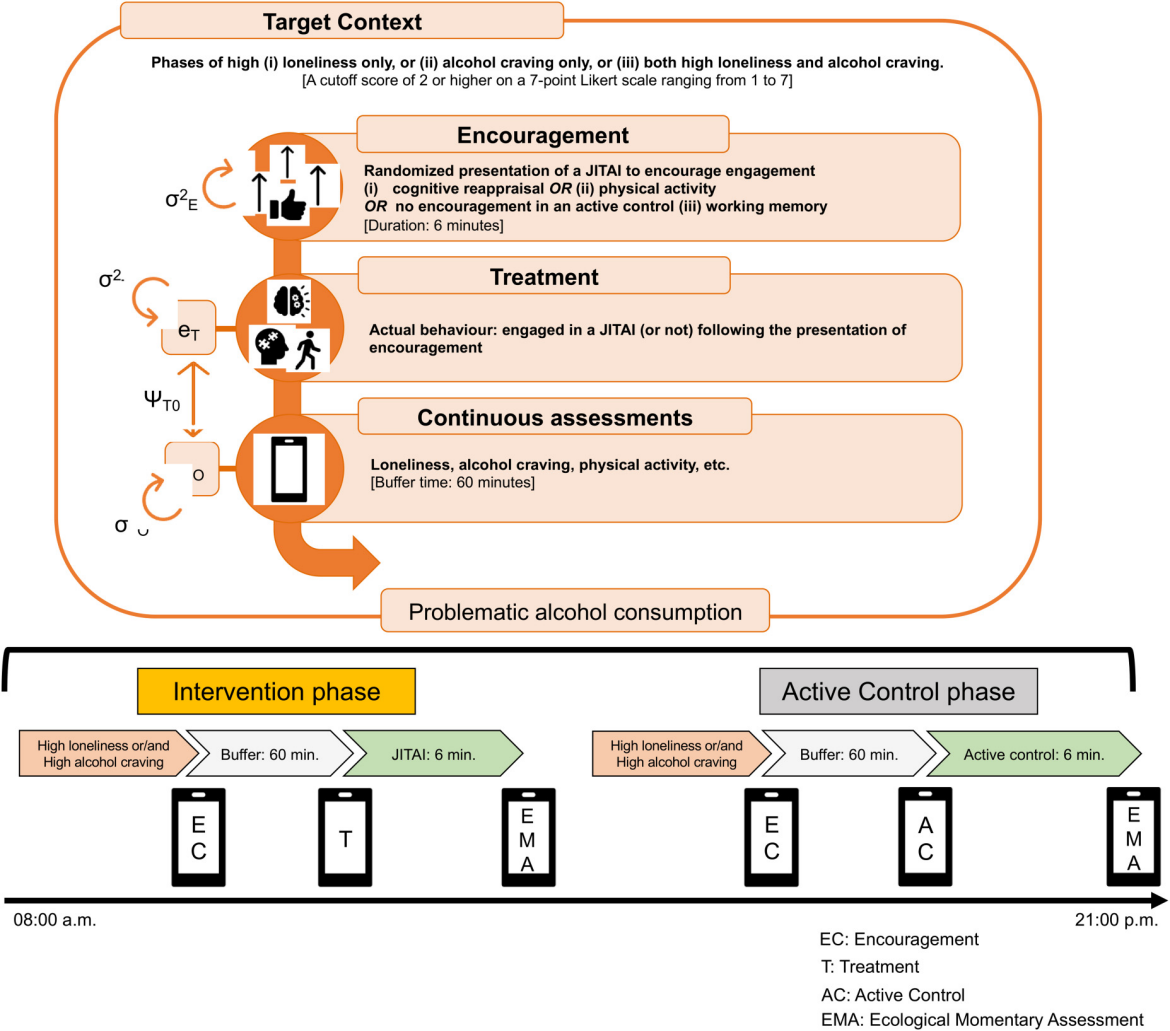
Overview of the within-person-encouragement-design specifications.

Randomized encouragement to engage in one of the abovementioned interventions is triggered through *movisensXS*, particularly in contexts where participants’ ratings indicate a high momentary level in at least one of the following target situations: i) high loneliness only, ii) high alcohol craving only, and iii) both high loneliness and craving alcohol. We will limit the number of triggers for interventions to a maximum of 3 per day. While two-third of these situations (*n* = 2), encouragement to engage in an intervention will be provided, the remaining situation (*n* = 1) serve as control condition without encouragement. Loneliness and alcohol craving are detected in real-time via analyses of the participants’ e-diary ratings, and physical activity is monitored via a sensor attached to the participants’ hip (*movisens* move-4), which is connected to the smartphone via Bluetooth low energy for data transfer and real-time analyses. To increase compliance, we will implement a “buffer time” of up to 60 min (see Figure 5).

### Outcome measures

Primary outcomes will be assessed at a momentary level, with (momentary assessments conducted eight times per day during the nonintervention phase and eight times per day during the intervention phase:

1. The score of a single-item to measure the direct loneliness^68^ on a Likert scale ranging from 1 to 7 and the three-item ULS-3 to measure the indirect loneliness in English^69^ and the respective validated German version^70^ on a VAS with the range 0 to 100.
2. The score of a single-item to measure direct craving^71^ using a Likert scale ranging from 1 to 7 and the five-item PACS to measure the indirect craving in English^72^ and the respective validated German version^73^ on a VAS with the range 0 to 100.

Secondary outcomes will be assessed at a momentary level, with momentary assessments conducted eight times per day during the nonintervention phase and eight times per day during the intervention phase:

1. The score of a 6-item Multidimensional Mood Questionnaire measured on a Likert scale ranging from 1 to 6^74^
2. The number and sort of standard drinks, German version^43^
3. The score of a modified version of the five-item Drug Effects Questionnaire-5 to measure subjective response to alcohol in English^75^ and in German^76,77^ using the VAS ranging from 0 to 100
4. The score of two modified single-items to assess control over drinking habits and feelings of loneliness^78^ measured on a VAS ranging from 0 to 100
5. The score of a two-item scale to assess event appraisal^79^ using a VAS which ranges from 0 to 100
6. The score of a three-item scale to measure momentary social contacts.^80,81^ This assessment includes the duration of social contacts in hours and minutes, as well as the quality of these contacts, rated on a VAS from 0 to 100
7. Movement Acceleration Intensity (MAI) steps and Euclidean Norm Minus One (ENMO)^82^ assessed automatically via a *movisens* Move4 wrist-sensor.^83^

Secondary outcomes measured on a daily basis (evening assessment conducted once per day):

1. The score of a modified four-item scale to measure motivation for alcohol consumption (social, coping, enhancement, and conformity)^43,84^ on a Likert scale from 1 to 5
2. The score from three items inquiring about drug usage other than alcohol, including usage and, if applicable, dosage of other drugs as well as cigarettes. The specific list includes: substances for sniffing (e.g. glue), tranquilizers or sedatives, stimulants (e.g. amphetamines, methamphetamines), hallucinogens (e.g. drug mushrooms, crack cocaine, cocaine), relevin, heroin, narcotics, ecstasy, ketamine or phencyclidine, GB or liquid ecstasy, anabolic steroids. This list is adapted from the National Survey on Drug Use And Health by the U.S. Department of Health and Human Services, Substance Abuse and Mental Health Services Administration^85^
3. The score of a modified two-item Patient Health Questionnaire-2^86^ on a Likert scale from 1 to 4
4. The score of a modified two-item Generalized Anxiety Disorder Scale-2^87^ on a Likert scale from 1 to 4
5. The score on a single item measuring the stressfulness of the day on a VAS ranging from 0 to 100 modified form Preston and coworkers^88^
6. The score of two 3-item Intention to Cope Questionnaires, modified to address loneliness^89^ and craving^90^ on a VAS from 0 to 100
7. The score of a 4-item Brief Impulsivity Scale^91^ on a Likert scale ranging from 1 to 5
8. The score of a single item considering limiting the alcohol consumption on the following day using a VAS from 0 to 100 modified from Howard and coworkers^92^
9. The score of a single item derived from the Reward Probability Index^93^ on a Likert scale ranging from 1 to 4
10. The score of a gamified stop-signal-task to measure response inhibition^50^
11. The score of a six-item scale to measure reappraisal using a VAS which ranges from 0 to 100^94^

To gain a deeper understanding of the interventions’ effects, we will monitor participants’ use of reappraisal during the physical activity intervention and vice versa. After each encouragement condition, we will calculate both the MAI and ENMO scores and assess reappraisal using the abovementioned six-item scale.^94^

Upon the participants’ return with the smartphone and wearable devices, we will conduct a qualitative semistructured interview, which will be audiorecorded and later transcribed. The following questions will be asked:

1. How feasible did you find the cognitive reappraisal, physical activity, gamified working memory task?
2. How engaged were you in the cognitive reappraisal, physical activity, gamified working memory task?
3. How acceptable did you find cognitive reappraisal, physical activity, gamified working memory task?
4. What did you find most frustrating or annoying about the app?
5. How could the study be improved?
6. What did you find most frustrating or annoying about the study in general?
7. Did your participation in the study (cognitive reappraisal, physical activity, gamified working memory task) help reduce your alcohol consumption? If so, how?
8. Did your participation in the study help reduce your feelings of loneliness? If so, how?
9. Did your participation in the study help reduce your craving for alcohol? If so, how?
10. How honestly did you complete the queries and interventions (cognitive reappraisal, physical activity, gamified working memory task)?

### Data analysis

We will analyze the resulting ILD using structural equation and multilevel modeling (MLM) with *R and Mplus*. The procedure including data preprocessing, analysis, and modeling is more detailed below. To preprocess EMA data, we will follow established state-of-the-art procedures detailed in methods guidelines.^12^ Physical activity data will be preprocessed by computing established metrics such as MAI and merged with the e-diary data (software Data Merger, *movisens* GmbH, Karlsruhe).

To assess the effectiveness of our interventions (i.e. cognitive reappraisal and physical activity) in reducing loneliness and cravings, we will employ instrumental variable estimation and build two-level structural equation models for path analyses,^21^ illustrated in Figure 5. On the first level (Level 1), we will estimate repeated measurements within subjects. In the path analyses, we will model (i) direct effects of the instrumental variable (encouragement for intervention) on the treatment (cognitive reappraisal and physical activity); (ii) the treatment effect on the target/outcome variables (i.e. loneliness or/and alcohol craving); and (iii) the residual (co-)variances (σ^2^_T_, σ^2^_0_, ψ_T0_) of the treatment (е_T_) and outcome (е_O_) on the within-subject level. On the second level (Level 2), we will estimate between subject differences in means, intercepts, and regression coefficients as random effects. Beyond two-level structural equation models, we will use established MLM procedures to test for within-subject changes in alcohol consumption and for pre–post differences in both mean levels of alcohol-use triggers (i.e. loneliness or/and alcohol craving).

For nonsystematic missing data,^47^ we will impute the missing values using the standard procedure.^95^ Specifically, we will employ the R package Multiple Imputation via Chained Equations to handle missing data through an iterative predictive modeling process.^14^ For the MLM and multilevel structural equation modeling analysis, we will employ the default FIML/MCM procedure in *Mplus*. In addition, we plan to explore the effects of reappraisal and physical activity in comparison to a no-intervention condition within a subsample of participants. The qualitative data will be analyzed using thematic analysis with the software MAXQDA. Our general approach involves identifying, analyzing, and reporting key themes. Specifically, we will read and reread the data to gain a deep understanding, generate initial codes, and group these codes into potential themes. We will then refine and review the themes to ensure they accurately reflect the data. Each theme will be clearly defined and named, and we will integrate the themes with relevant data extracts for reporting purposes.

## Expected results

We anticipate that our platform will demonstrate feasibility in administering both cognitive reappraisal and physical activity interventions, which are expected to reduce cravings and loneliness. In addition, it will facilitate the integration of experimental manipulation into participants’ daily routines, enabling effective testing of causal relationships. We expect that both cognitive reappraisal and physical activity interventions will decrease momentary loneliness (H1a) or/and alcohol craving (H1b). Furthermore, we posit that decreases in momentary loneliness and alcohol craving will be associated with a reduction in real-life alcohol consumption (H2).

## Discussion

In this study, we will integrate real-time mechanism-based interventions for alcohol use while simultaneously collecting data on behavioral patterns and physical activity through a smartphone app and a wearable sensor. This study marks a pioneering mechanistic investigation aimed at understanding the underlying mechanisms of action of interventions and behavioral processes among individuals with problematic alcohol use in Germany. By employing real-time data collection and momentary sensing techniques, the study specifically targets the mechanisms underlying loneliness and craving in the context of alcohol consumption.

JITAIs can be particularly valuable in the treatment of AUD, especially given the significant gaps in face-to-face treatment services.^96,97^ Potential applications of this JITAI include providing transitional support for individuals with AUD awaiting formal psychotherapeutic treatment and offering an alternative for those with milder AUD who may not require intensive psychotherapy.

Despite its potential, our current study has several limitations. Firstly, compared to an in-person intervention, we have less control over participants’ engagement with the intervention. To address this issue, we plan to closely monitor participant engagement and compliance throughout the study. In addition, the timing of a JITAI is crucial for triggering the intervention at the right moment.^19^ There is a risk that we may not assess craving or loneliness at the optimal times. Nonetheless, we must find a balance between the intensity of our sampling and the burden placed on participants.

The study will adopt multiple perspectives. First, it seeks to assess generalizability to other SUDs in future studies. Second, it aims to implement efficient digital interventions. For example, we can implement a progress indicator—a rotating display that shows the status of participants’ intervention progress in future work. Participants may accumulate points to reach modular intervention levels, displayed via sophisticated graphs such as growing trees with an increasing number of branches and leaves. Gamified progress indicators (scores and levels) can be implemented via the TherapyDesigner and TherapyBuilder (both movisens GmbH, Germany). Third, alternative causes and interventions for SUD will be explored. We can extend our approach to possible alternative causes of SUD and respective intervention strategies in the general populations. Importantly, insights into temporal directionality and digital intervention adherence gained from the current study will inform the development of the first evidence-based tailored and expedient JITAIs in SUD. Fourth, transferability and scalability to other sociocultural contexts can be explored in future studies. Ultimately, we will develop an in-silico model of losing and regaining control in alcohol use based on variational autoencoders and provide evidence-based treatment options across cultures.

## Conclusions

Our real-life intervention platform is designed as a robust tool for discerning the directionality of effects from time series data related to everyday influences on SUD. This study may pave the way for low-threshold prevention, clinical treatment, and therapy tailored to the diverse contexts of SUD, thereby facilitating rapid advancements in SUD healthcare. Moreover, our findings hold the potential to establish an insights framework for developing effective strategies to manage substance use risks in everyday life.
